# Help Wanted and Received

**Published:** 1890-09

**Authors:** Edward T. Balch

**Affiliations:** Summerland, Cal.


### HELP WANTED AND RECEIVED.

EDITORS OF THE HOMŒOPATHIC PHYSICIAN:—My call for *help* in the June number of your journal, page 286, has elicited very many gratifying responses—indeed, from the Pacific to the Atlantic, from England, from Germany even, help in time of need has been freely tendered in the suggestions of Dr. Geo. H. Clark, of Philadelphia; Prof. A. McNeil, of San Francisco, and Dr. E. W. Berridge, of London, England. I gave the case, on June 28th, a dose of Lac-can.$^{50m}$ dry on tongue, with the pleasing result that, in twenty-four hours, the child became more lively and playful, all abnormal sensations have disappeared, and this day (July 20th) appears well in every respect.

The following gentlemen also have furnished most valuable advice: Dr. Alex. Villers, Dresden, Germany; Dr. J. T. Martin, Woodland, California; Dr. E. H. Holbrook, Baltimore, Md.; Dr. H. M. Kearney, De Soto, Mo.

To each and every one allow me, through your valuable pages, to tender my most sincere and heartfelt thanks.

Fraternally,

SUMMERLAND, CAL. EDWARD T. BALCH, M. D.

